# A Review of Gingival Recession and the Surgical Managements According to Their Classification and Etiologic Backgrounds: A Clinical Case Study

**DOI:** 10.1155/2024/5510846

**Published:** 2024-01-31

**Authors:** Dler Ali Khursheed, Faraedon Mostafa Zardawi, Awder Nuree Arf

**Affiliations:** ^1^Department of Periodontology, College of Dentistry, University of Sulaimani, Sulaymaniyah, Kurdistan Region, Iraq; ^2^Faculty of Dentistry, Qaiwan International University, Sulaymaniyah, Kurdistan Region, Iraq; ^3^Department of Pedodontics, Orthodontics and Preventive Dentistry, College of Dentistry, University of Sulaimani, Sulaymaniyah, Kurdistan Region, Iraq

## Abstract

Mucogingival deformities are a group of defects that occur around the cervical area of the teeth. Gingival recession is the most common type of these deformities. It might happen separately or with other related deformities like thin gingival biotypes, shallow vestibule, high frenal attachment, and cervical dental steps. Recent classification of mucogingival deformity matrix has collectively grouped gingival recession types with other mucogingival deformities and dental steps to establish the proper diagnosis and evaluate surgical/restorative management and prognosis of the treatment outcomes. The following case presentations have presented with a technical review, clinical evaluation, and surgical and/or restorative treatment according to the recent matrix.

## 1. Introduction

The free gingival margin course runs parallel to the cementoenamel junction (CEJ) [1]. The gingival margin is located at different levels of the clinical crown according to the stage of tooth eruption; it could be either in an active or passive eruption stage [2], and therefore, the fundamental and the static landmark for the presence and absence of gingival recession (GR) is the CEJ.

Gingival recession could be defined as the location of marginal periodontal tissues (gingival margin) apical to the CEJ [3], which is associated with the loss of periodontal apparatus, including gingiva, periodontal ligament, and root cementum, and this often exposes the recessed root surface to the oral environments [4, 5]. As a result of periodontal attachment loss, often periodontal recession is used interchangeably with GR. In the 2017 World Workshop on the Classification of Periodontal and Peri-Implant Diseases and Conditions, the GR was not defined as periodontitis despite the condition designating clinical attachment loss [5]. The primary two causes of GR are biofilm-induced inflammatory process and physical gingival wear that occur in populations with low and high standards of oral hygiene, respectively [6]. GR could be presented either in a localized form or a generalized form; further, it could be presented either on the labial or lingual surfaces and/or interproximal areas. The recent classification of GR is based on the relationship between the amount of recession at the proximal and buccal/lingual attachment loss and their severity [7].

In addition to the severity of the GR, factors such as gingival biotypes should be considered for treatment planning of gingival tissue deformities. Gingival biotypes can be classified as thin scalloped, thick flat, and thick scalloped. These biotypes are characterized based firstly on gingival keratinized tissue width, secondly on the bone morphotype, and thirdly on the tooth dimensions: crown length/crown width [8, 9]. However, interdental relationships like the presence of crowding and spacing can alter the biotype records. Patients with a thin tissue biotype have a more delicate type of gingiva that will be more susceptible to developing recession, especially when they are subjected to either mechanical trauma and/or inflammation [10]. Absence of keratinized tissue is considered a risk factor for the development of GR; on the other hand, the presence of a sufficient amount of attached gingiva is essential for the maintenance of the gingival health [5, 11]. It was accredited that the presence of ≥2 mm of a width of keratinized gingiva is beneficial for maintaining the gingival health in the presence of subgingival restorations. Therefore, gingival augmentation may be recommended at sites with minimal or no gingival tissue, particularly when the margin of the restoration is extended subgingivally.

In the current mucogingival deformity classification, in addition to gingival biotypes, detection of CEJ, the presence and absence of steps due to cervical tooth wear have also been considered for planning the mucogingival surgery [5]. The position of the CEJ level can also be affected by the vertical positions of the tooth with the adjacent teeth. Teeth with more recessions (attachment loss) could be prone to overeruption, drifting, and tipping.

Correspondingly, the two supplementary factors that should also be considered in the treatment of GR are vestibular depth and muscle/frenal aberrant positions. A shallow vestibule hinders the plaque control methods and oral hygiene performance, which leads to incomplete removal of plaque deposits. The muscular traction provided by the vestibule may enhance GR. The presence of a high labial frenal attachment can be an additional factor in the progression of GR [12]. The aim of this complementary case presentation was to describe four different GR cases with different etiologic backgrounds that were associated with other dental and mucogingival deformities and to designate the surgical procedures for gingival augmentation over the recessed roots and the gingival tissues around the recession to prevent further future recession, through increasing the width and thickness of the gingiva, increasing the vestibular depth and elimination of muscle/frenal tissues to enhance oral hygiene methods performed at the area of the recession [5, 10, 13].

All the patients who participated in the current study signed a consent for their participations in the current study, and the manuscript obtained ethical approval from the ethical committee of the College of Dentistry/University of Sulaimani (N21 at July 10th, 2019).

## 2. Case One

**Figure 1:**
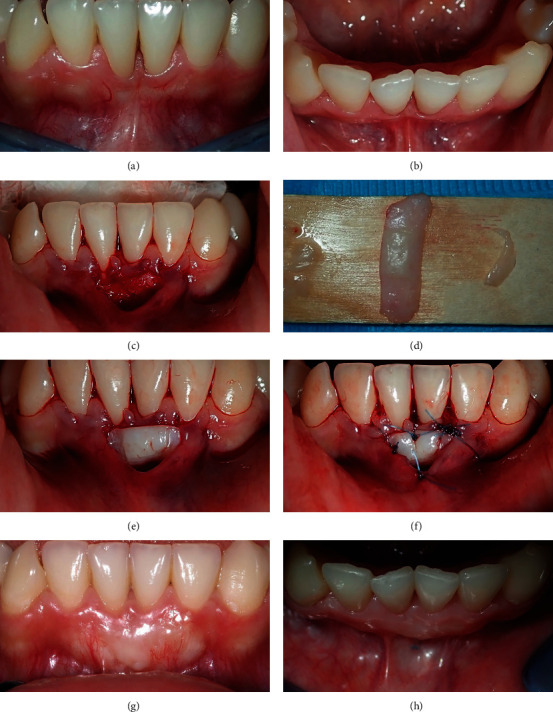
The two lower central incisors show false gingival recession with thin and narrow interdental papilla (a), the occlusal view shows the mesial rotation of both central incisors and obvious frenal attachment (b), the flap was raised with pouching laterally and apically (c), FGG was deepithelialized on lateral ends (d), the graft was placed over the recession and deepithelialized ends placed under the pouch (e), the graft was sutured to the recipient tissue margins (f), and the area is covered with healthy, thick, and keratinized tissue (g, h).

An 18-year-old female patient attended with interproximal tissue loss and mild mesial rotation of both lower central incisors. Facially, there was a discrepancy in the level of the marginal gingiva of both central incisors with slight gingival recession; both central incisors showed a more apical level of the marginal gingiva than the left and right lateral incisors, Similarly, the interdental papilla between the central incisors showed a reduced height compared to adjacent papillae, exposing the roots on the mesial sides of the central incisors. At the free gingival margin, there was a lack of keratinized gingiva of the labial gingival tissue over the crowns of the two central incisors, whereas a wide band of attached keratinized gingiva was seen on lateral incisors; further, none of the four incisors were proclined labially. The labial vestibule was shallower with pronounced frenum on the gingival tissue against central incisors compared to the lateral incisors. The CEJs were covered with gingiva, and there was no sign of steps at the cervical area (Figures 1(a) and 1(b)). It was impossible to apply RT for the treatment of this case because the buccal recession did not extend apical to the CEJ (Table 1).

### 2.1. Recession Management

A partial thickness flap was performed just apical to the gingival margin of the central incisors and extended laterally and apically, and the frenal fibers were dissected. The pouch was prepared with a tunneling instrument, and then, it was extended and raised laterally (Figure 1(c)).

A free gingival graft of about 1 mm thickness was taken from the molar area in the palate, and its length and width are taken according to the severity of the recession. Hemostatic sponge gauze was placed and sutured to the donor area. The graft was deepithelialized on both sides, while the epithelium remained intact in the middle part of the graft (Figure 1(d)). The flap was then positioned over the recipient area in such a way that the deepithelialized ends of the graft were placed under the pouch, and the epithelialized central portion of the graft was placed over the recession area. The coronal part of the graft pulled the gingival margin tags coronally (Figure 1(e)). The graft then was secured and immobilized with interrupted sutures (Figure 1(f)). Postoperative antibiotics (amoxiclav 1 gm twice daily for 5 days) with nonsteroidal anti-inflammatory (Ibuprofen 400 mg) were prescribed for the patients, and they were kept on chlorhexidine mouthwash (0.12% twice daily) for two weeks.

### 2.2. Result

The results showed that the recession-like area of the crowns of the two central incisors was totally covered. Labially, the gingival level discrepancy between the central incisors and lateral incisors was managed and had almost disappeared. The interdental papilla had also improved in consistency from a thin delicate gingival to a thick triangle that filled the interdental space (Figure 1(g)). Twenty-four-month postoperative follow-up shows the increase in the thickness of the keratinized gingiva (Figure 1(h)). The gingiva at the treatment site reflects a whitish color compared to the color of the gingiva at the adjacent areas.

## 3. Case Two

**Figure 2:**
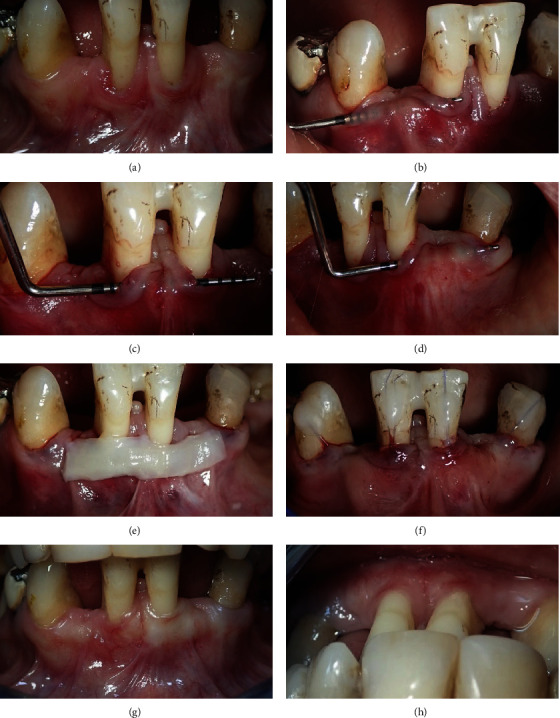
Two lower central incisors show RT2 recession with missing adjacent lateral incisors (a), tissue tunneling was performed up to the midline of both canines (b–d), deepethialized CT was examined over the recession and edentulous area (e), the CTG was fixed with sutures over the teeth (f), and partial root coverage with thick tissue was achieved after 3 months (g, h).

A 53-year-old female attended with periodontal attachment loss on lower central incisors with GR. There was a narrow band of keratinized tissue apical to the free gingival margin, and no mobility was detected at either of the central incisors (Figure 2(a)). The two adjacent laterals were extracted due to periodontitis, and the facial and proximal tissues were approximately showing similar levels of attachment loss from CEJ. The patient was referred for treatment of the recession and ridge augmentation before the construction of fixed prosthodontics. The CEJ was observed on both teeth facially and interproximally. No root caries or noncarious lesions were detected on clinical examination of the teeth. The central incisors showed a slight lateral drifting with a high level of the remaining interdental papilla. The condition could be categorized as RT2 on the reduced periodontium. Besides, there was a sufficient vestibular depth at the area of the labial frenum (Table 1).

### 3.1. Recession Management

The tunneling procedure at the recession area was performed with a Hu-Friedy tunneling instrument (TKN1 #1 HDL #6); a partial thickness flap was performed and extended apically and laterally halfway to the mesial aspect of right and left canines. Coronally, the tunneling extended to the level of the alveolar ridges of the lateral incisor edentulous areas (Figures 2(b)–2(d)). A long free gingival graft of 1 mm thickness was harvested from the palate and completely deepithelialized with a 15c blade (Figure 2(e)). The graft was placed under the tunneling pouch and stretched laterally by the sutures, as previously described [14] and fixed to the facial aspects of the left and right canines with composite, and then, the graft was advanced coronally on the labial surface of the crowns (Figure 2(f)). Postoperation medications and instruction were given to the patient.

### 3.2. Result

Partial root coverage was achieved after 3 months, with increased gingival thickness on both central incisors and on the edentulous area. The gingival margins on the central incisors were now very close to the coronal level of the edentulous area's height (Figures 2(g) and 2(h)).

## 4. Case 3

**Figure 3:**
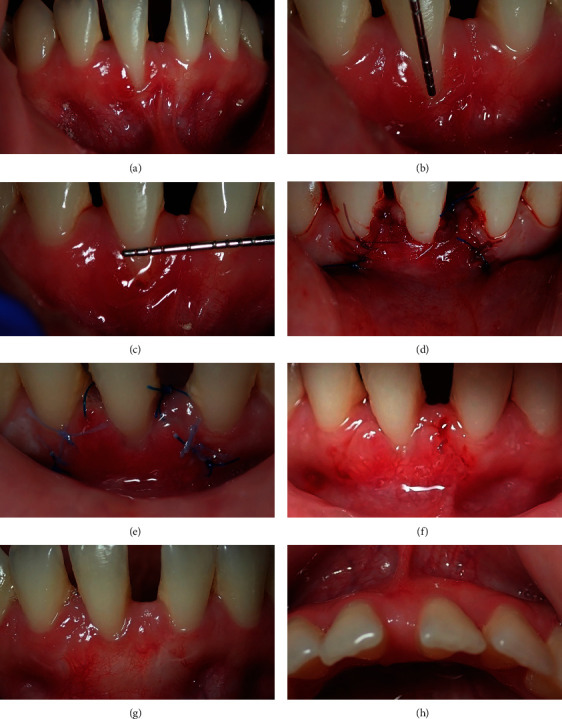
Clinical image shows RT2 with no keratinized tissue around the recession (a), the recession length and width are 3 mm and 2 mm, respectively (b, c), coronally advanced flap with CTG (d), day 3 shows inflammation around the surgical area (e), day 14 shows shrinkage on the surgical area (f), postoperative labial and occlusal views show proper root coverage with proper thickness (g, h).

A 22-year-old female was referred with GR on tooth number #31 with spacing between the teeth. The tooth had drifted laterally, and there was a deep vestibule, high frenal attachment, and a thin gingival biotype, with no keratinized gingival tissue on the tooth that was revealing a recession (Figure 3(a)). The recession dimensions were 3 mm high and 2 mm wide, and the root was exposed interproximally, displaying CEJ obviously at both interproximal and labial surfaces of the tooth with a flat interdental papilla (Figures 3(b) and 3(c)). Therefore, this RT2 was associated with CEJ/Step A- and deep vestibule. Postoperative medications and instructions were given to the patient (Table 1).

### 4.1. Recession Management

Coronally advanced flap was performed with CTG. A horizontal incision was made 2 mm apical to the interdental papillae, combined with two vertical incisions to raise a partial thickness flap adjacent to the recession, and the papillae were deepithelialized with a blade. Then, the alveolar mucosa was undermined, and the frenal fibers were dissected. A free gingival graft of 1 mm thickness was harvested from the palate and deepithelialized with a 15c blade. The graft was then placed to cover the recession; the graft was fixed to the interdental papillae with an absorbable suture. The flap was placed over the graft and fixed with two sling sutures, and the vertical incisions were sutured by interrupted suture stitches (Figure 3(d)).

### 4.2. Result

The patient was followed on days 3 and 14 and at 6 months. After 6 months, full coverage of the recessed root surface was achieved with a thick gingival tissue, as the occlusal view shows (Figures 3(e)–3(h)).

## 5. Case 4

**Figure 4:**
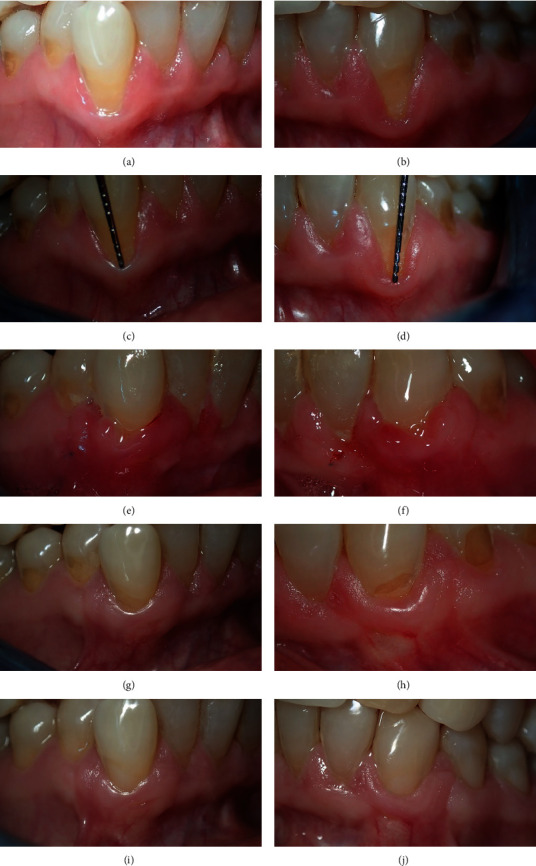
Both canines show RT1 with steps (a, b), and clinically about 4 mm and 5 mm recession on #33 and #43, respectively (c, d); day 14 shows inflammation around the surgical areas (e, f); after three months, healthy and keratinized tissue can be seen with partial exposure of the steps (g, h), and after 6 months, there is still a healthy and stable gingiva present around the composite filling (i, j).

A 42-year-old female patient was referred with a chief complaint of hypersensitivity and tooth elongation of both #33 and #43 (Figures 4(a) and 4(b)). On clinical examination, RT1 of 4 mm and 5 mm recession was detected on #33 and #43, respectively (Figures 4(c) and 4(d)). Furthermore, both recessed roots clinically showed invisible CEJ with noncarious cervical tooth wear B+. Both teeth were labially proclined, especially the #33. The probing depth was 1 mm, and there was a band of 2 mm keratinized tissue around the recessions, with thick and wide interdental gingival papillae mesially and distally. Further, at both teeth, there was sufficient vestibular depth apical to the recession (Table 1).

### 5.1. Recession Management

Subpapillary horizontal incisions were made on both sides, with two vertical incisions lateral to them; a partial thickness flap was performed to the alveolar crest and then followed by a full thickness flap extended to the vestibular fornix. The gingival papillae were deepithelialized, and the flap was raised and fixed to the papillae with a sling suture technique. The vertical incisions were sutured with interrupted 04 nylon sutures. After 3 months, the cervical tooth wear was treated with composite fillings.

### 5.2. Result

After 2 weeks, the flaps were covering the recession completely (Figures 4(e) and 4(f)), and 3 months later, the gingiva looked healthy and stabilized (Figures 4(g) and 4(h)). After 6 months, satisfactory root coverage was achieved with a good quality of gingival tissue. The tissue was very similar in appearance to the original (Figures 4(i) and 4(j)).

## 6. Discussion

Surgical management of GR is aimed at achieving complete root coverage to improve esthetics and function and to enhance the maintenance of periodontal health [15]. The absence of keratinized tissue may enhance GR, especially when the patient has been nominated to have an orthodontic appliance or prosthodontics treatment. Therefore, gingival augmentation is an essential preventive measure before the patient undergoes any of the above-mentioned treatments. These procedures may be accomplished simultaneously by releasing the muscle fibers and increasing the vestibular depth and improving the gingival biotype. This will enhance the patient's oral hygiene measures and stabilize the newly augmented gingival tissue at the area of the recession; further, it enables the status of the cervical lesion to be identified accordingly.

Recording interproximal attachment loss is considered an essential step in the recent classification of GR. It is also considered an important step for designing the exact treatment approach for every individual case and defining the prognosis of the treatment outcome.

A wide variety of mucogingival deformities could be detected, including a single or multiple GR and a single recession with a high frenal/muscular attachment and shallow vestibular depth. Simultaneous management of these deformities improves the long-term stability of the gingival status. Further, releasing the flap tension during the surgical procedure improves long-term prognosis [16]; for instance, in the case of #1, the loss of keratinized tissue was associated with shallow vestibule and high frenal attachment with firm underlying muscle fibers. Dissecting the muscle fibers and placement of FGG simultaneously with the partial tunneling technique are important steps to accomplish in applying a tensionless flap and increasing the source of blood supply to the graft. After an interval of two years, sufficient vestibular depth and a favorable thickness and amount of connective tissue were observed in the area. Rasperini et al. have related a variety of factors to the clinical outcome of surgical management of gingival tissue deformity and long-term stability of the treatment, such as severity of the condition, surgical technique, and operator's skill [17].

The esthetic outcomes, such as color, thickness of the gingival tissue, and interdental papillary appearance, also needed to be considered, especially in cases of recession associated with lost and thin interdental papilla.

In the current case series, the following case presentations were selected based on different circumstances with a variety of clinical significances, not merely for halting recessions and preventing further recession from occurring, but also to enhance the future restorative treatment and to achieve a stable gingival relationship with the margin of the restoration by improving the patient's plaque control method at the dentogingival area where the margin of the restoration is located.

In the first scenario, the condition obviously appears as GR on #21 and #41; however, no recession was recorded according to the criteria of recession detection, and this diagnostic point of “cases of passive or false GR” has not yet been defined even in the most recent classification of recession. Therefore, in these situations, the stage of eruption, whether active or passive eruption, should be regarded while detecting recession. Case #1 in the current study had lost keratinized tissue accompanied by a shallow vestibule and was more vulnerable to further recession. A free gingival graft is frequently used to increase keratinized tissue and increase vestibular depth [18, 19], as the tunneling procedure increases blood supply from both overlying and underlying tissues. Since the graft was planned to be placed over the enamel and the thin interdental papillae, partial deepithelizing was performed of the lateral parts of the graft to be placed beneath the flap, and at the center, the epithelized part had covered the recessed root surface. The two-year follow-up revealed healthy gingiva and thick keratinized tissue covering the crowns. The previously thin interdental gingival tissue and the esthetic had also improved.

In the current case series, the second scenario presented recession associated with a thin gingival biotype in the reduced periodontium. To the best of our knowledge, this is the first study to describe the treatment of GR with reduced periodontium. The consequences of the previous periodontitis had led to a circumferential exposure of the root surfaces [20]. Although Chambrone and Avila-Ortiz proposed a novel classification for gingival tissue deformities and recession [15], the study described this kind of gingival tissue deformity, which could be observed in daily practice with cases of GR. This recession can be defined as GRD-II or RT2. Buccal or lingual GR defects present with adjacent interproximal attachment and bone loss, where the midbuccal/midlingual clinical attachment level is apical to the interproximal bone level. Increasing gingival thickness is important for prerestorative treatments like crown and bridges or dental implant placement [21]. In addition to increasing gingival thickness around the recessions, the thickness of adjacent edentulous ridges had increased too. Raising the tissue to the level of CEJ is impossible in this RT2 [15]. Therefore, while it was not possible to restore the interdental papilla to the normal level, the height of the residual ridge was increased, and the normal gingival color was restored by tunneling procedure, with none of the color discrepancy that might happen with orthokeratinized FGG.

Similarly, the third case was associated with a very thin gingival phenotype with no keratinized tissue. Therefore, it is clinically of significant value to increase gingival thickness with the root coverage procedure. The presence of a deep vestibule would enhance raising the flap over the CTG passively. Successful root coverage with improved gingival phenotype was achieved by Zucchelli et al., for the treatment of deep gingival recession affecting the mandibular incisors using an advanced coronal flap over a free connective tissue graft [22]. After six months, complete root coverage with proper gingival thickness was achieved to provide a stable gingival status for undertaking the orthodontic treatment safely.

In the last case of the present report, the recession was associated with a thin gingival biotype and cervical step. However, the presence of about 2 mm of keratinized tissues around the recessions with proper vestibular depth was sufficient to undertake a coronally advanced flap to cover the recession. This would also eliminate the second surgical procedure for taking a palatal graft.

After 3 months of a healing period and restoration of the cervical tooth wear, a proper and stable gingival health was maintained. CEJ is the hallmark reference for determining the rate of root coverage, in the current situation; however, it is not possible to claim that full root coverage was achieved since the procedure included a step due to cervical tooth wear, which frequently obscured the detection of CEJ as an anatomical landmark used for recognition of CEJ.

**Figure 5:**
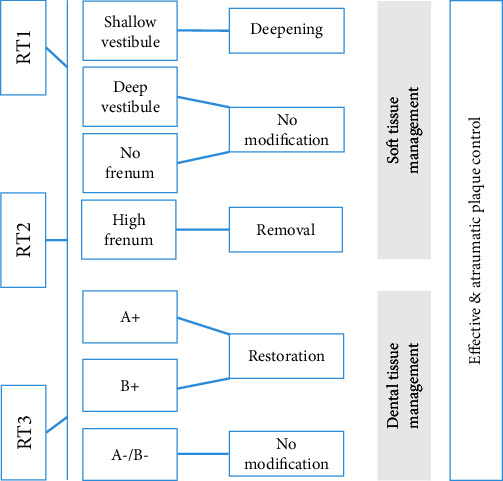
Mucogingival assessment and management according to the 2017 World Workshop on the Classification of Periodontal and Peri-Implant Diseases and Conditions.

Therefore, management of mucogingival deformities including GR should be based on a thorough understanding and inspection of the anatomical, etiological background at the site and around the recession and establishing an appropriate classification stage of each individual condition accordingly, in order to obtain an optimal and a stable mucogingival relationship and to achieve stable long-term health of the periodontium at the site of recession (Figure 5).

It is worthy to mention the presence of a 2 mm band of keratinized tissue of which 1 mm attached gingiva will be sufficient to maintain optimal gingival health. However, the presence of the above-mentioned deformities and improper oral hygiene control may prone this delicate tissue to recession by gingival inflammation. In this situation, surgical intervention, to eliminate these aberrant mucogingival tissues and increase the connective tissues, will facilitate proper oral hygiene measures and halt destructive inflammation too [23].

The limitation of the current study is the number of cases that have been presented that could not cover all the aspects of the classification of mucogingival deformities; therefore, further study is recommended with the higher number of cases.

## 7. Conclusion

The clinical case series concludes that a satisfactory result could be achieved for covering different types of recession surgically by a thorough understanding of their classification, clinical presentation, etiologic backgrounds, and appropriate surgical procedure selection.

## Figures and Tables

**Table 1:** Severity of gingival recessions and their relations with other mucogingival deformities and cervical dental conditions.

Gingival site			Tooth site		
	REC depth	GT	KTW	CEJ (A/B)	Step (+/-)
*Case 1*					
No recession	0	Thin	1<	?	?
RT1	?				
RT2	?				
Rt3	?				
*Case 2*					
No recession		Thin	1<	A	—
RT1					
RT2					
Rt3					
*Case 3*					
No recession	3 mm	Thin	0	A	—
RT1					
RT2					
Rt3					
*Case 4*					
No recession					+, +
RT1	4, 5	Thick, scalloped	2	B	
RT2					
Rt3					

## Data Availability

All required raw data are available at request, and besides, most data are included in the manuscript.
